# Effects of Dietary Rutin Supplementation on the Intestinal Morphology, Antioxidant Capacity, Immunity, and Microbiota of Aged Laying Hens

**DOI:** 10.3390/antiox11091843

**Published:** 2022-09-19

**Authors:** Hengzhi Li, Yunfeng Gu, Rui Jin, Qingfen He, Yanmin Zhou

**Affiliations:** College of Animal Science and Technology, Nanjing Agricultural University, Nanjing 210095, China

**Keywords:** rutin, aged laying hens, intestinal morphology, antioxidant capacity, immunity, microbiota

## Abstract

This research investigated the effects of dietary rutin supplementation on the intestinal morphology, antioxidant capacity, immunity, and microbiota of aged laying hens. The results showed that 500 mg/kg rutin supplementation increased the villus height of jejunum (*P* < 0.05). Rutin affected the immune system of the ileum and the jejunum. In the jejunum, a diet with 500 mg/kg rutin supplementation enhanced secretory immunoglobulin A (sIgA) and reduced tumor necrosis factor-α (TNF-α) levels (*P* < 0.05). A diet with 1000 mg/kg rutin supplementation increased jejunal sIgA, immunologlobulin M (IgM), and interleukin-4 (IL-4) levels while decreasing interleukin-1β (IL-1β), TNF-α, and interferon-γ (IFN-γ) levels (*P* < 0.05). Meanwhile, a diet with 500 mg/kg rutin increased sIgA, immunologlobulin G (IgG), IgM, and interleukin-10 (IL-10) levels and reduced TNF-α and IFN-γ levels in the ileum (*P* < 0.05). In the ileum, a diet with 1000 mg/kg rutin supplementation raised sIgA, IgG, IgM, IL-4, and IL-10 levels while decreasing IL-1β, TNF-α, and IFN-γ levels (*P* < 0.05). At the family level, a diet with 500 mg/kg rutin supplementation raised the relative abundance of Monoglobaceae and decreased the relative abundance of Eubacteriaceae (*P* < 0.05) compared to the control group. In the 1000 mg/kg rutin group, the relative abundance of Lactobacillaceae and Unclassified Coriobacteriale was considerably lower and the relative abundance of Monoglobaceae was higher than the control group (*P* < 0.05). This study showed that a diet with rutin supplementation can improve the intestinal health of aged laying hens, and the mechanism is related to improving the intestinal morphology and intestinal immune status, and regulating the intestinal microbes.

## 1. Introduction

Aging is a complicated process that involves morphological and metabolic changes in individual cells as well as the whole organism. The oxidative stress theory is one of the most prevalent ideas for how aging happens at the molecular level [1]. During the aging process in laying hens, harmful reactive oxygen species (ROS) are gradually produced, resulting in oxidative stress damage, including a decrease in liver antioxidant capacity [2,3], changes in intestinal oxidative state [4], and the aging of reproductive organs [5,6], which lead to a decline in egg production performance. Oxidative stress reaches its peak when the hen is about 65 to 70 weeks old [7]. After 75 weeks of age, laying hens’ performance often declines significantly [8], which is connected with oxidative damage and lower immunity [9,10,11,12,13]. The gut, as a key organ of diet digestion, absorption, and transformation, is easily impacted by oxidants and antioxidants in diet, resulting in redox balance shifts. Oxidative stress is a major contributor to the onset of inflammatory disorders [14]. Furthermore, intestinal inflammation decreases nutritional absorption and egg production, and inflammatory cytokines in turn can cause oxidative stress [15]. Changes in intestinal morphology, antioxidant status, and inflammation lead to poor intestinal health, which is one of the key causes of poor laying performance [16]. Changing components of the diet can quickly affect the makeup and activity of the gut microbiota [17], which plays a significant role in the development of the immune system in mammals and poultry, as well as maintaining immune system homeostasis [18,19].

Polyphenols, which are critical micronutrients in the diet, are one of the most abundant and widespread groups in the plant world, with over 8000 polyphenolic chemicals discovered. Polyphenols (phenolic acids, flavonoids, stilbenes, and lignans) have been extensively explored in recent decades due to their numerous health advantages [20,21]. Polyphenols have anti-inflammatory, antibacterial, and antioxidant activities that can affect egg production and health in older laying hens by improving gut integrity and function, lowering inflammation, or modifying microbial communities [22,23]. In older laying hens, resveratrol administration improved performance, lipid-related markers, and antioxidant activity [24]. Tea polyphenols can improve serum and liver antioxidant capability while increasing egg production [25,26,27]. Flavonoids have been used as feed additive to promote laying hens health and productive performance [28]. Rutin is a flavonoid that is found in abundance in plants. Rutin has a wide range of pharmacological effects on biological systems, including analgesic, anti-inflammatory, and anti-arthritis properties [29,30,31]. Rutin supplementation at doses of 0.50 and 1.00 g/kg substantially increased superoxide dismutase (SOD), catalase (CAT), and glutathione peroxidase (GSH-Px) activity and decreased malondialdehyde (MDA) concentrations in serum, showing rutin’s potential to transport electrons, activate antioxidant enzymes, and thereby reduce oxidative stress [32]. Among the immunological parameters studied, lysozyme activity, nitric oxide concentrations, and IgM synthesis were considerably greater in rutin-fed birds than in control birds; however, rutin at any quantity had no influence on IgG and IgA concentrations or lymphoid organ weight [33]. Previous studies suggested that rutin is a promising feed additive for broilers. Considering that aged laying hens suffer from significant oxidative damage and lower immunity, the use of antioxidants is crucial; however, there is limited study on the effects of antioxidant rutin on aging laying hens. The purpose of this research was to investigate rutin’s antioxidant capacity, immunomodulatory effects, and gut microbiome impacts in an aged laying hen.

## 2. Materials and Methods

### 2.1. Animals, Diets, and Experimental Design

All procedures were conducted under the guidelines of Nanjing Agricultural University Institutional Animal Care and Use Committee (Certification No.: SYXK(Su)2017-0007).

A total of 216 Hyline Brown aged laying hens (560 days of age) were obtained from a local commercial laying hens farm in this study. Laying hens were randomly divided into three treatments with six replicates per group. After a two-week adjustment, laying hens were separately fed with one of three kind of diets: basal diet; basal diet +500 mg/kg rutin; basal diet +1000 mg/kg rutin, and the trial lasted eight weeks. Rutin was provided by Jiangsu Bison Biotechnology Co., Ltd. (Taizhou, Jiangsu, China). The content of rutin was 96%, determined by high-performance liquid chromatography (HPLC) diffraction. These diets were formulated to meet or exceed the National Research Council (NRC, 1994) recommendation, and the composition and nutrient levels are shown in Table 1. Hens were free to drink water throughout the experiment and were exposed to a 16-h/8-h light/dark cycle.

### 2.2. Sample Collection

The small intestine was dissected and sampled on a refrigerated stainless-steel tray, clear of the mesentery. Middle sections of the jejunum and ileum from all hens were harvested, flushed several times with ice-cold phosphate-buffered saline (pH 7.4), fixed with 4% paraformaldehyde-PBS, and kept at 4 ${}^{{}^{\circ}}$C for evaluation of the mucosal morphology. The jejunum and ileum mucosas were quickly scraped and fully washed with ice-cold phosphatebuffered saline before being frozen in liquid nitrogen and stored at −80 ${}^{{}^{\circ}}$C, for intestinal antioxidant capacity and immune parameters, as well as mRNA expression analysis. Meanwhile, the contents of the cecum were collected aseptically, put in a centrifugal tube, quickly frozen in liquid nitrogen, and kept at −80 ${}^{{}^{\circ}}$C for microbiota analysis.

### 2.3. Intestinal Morphological Analysis

The middle sections of ileum and jejunum from all laying hens were removed from 4% paraformaldehyde-PBS and rinsed with water. Then, they were dehydrated with a series of different ethanol concentrations, cleared with xylene until saturation, and embedded with paraffin. A section of 5 μm was cut for histological analysis. The sections were deparaffinized and hydrated, and then stained with H&E. Villus height and crypt depth were measured.

### 2.4. Intestinal Antioxidant Capacity

The SOD activity and MDA, total antioxidant capacity (T-AOC) levels in jejunal mucosa and ileal mucosa were measured in accordance with the manufacturer’s instructions using a commercial kits (Nanjing Jiancheng Bioengineering Institute, Nanjing, China).

### 2.5. Intestinal Immune Parameters

Protein concentration was tested in the supernatant of the jejunum mucosa and ileum mucosa. Meanwhile, sIgA, IgG, IgM, IL-1β, TNF-α, IFN-γ, IL-4, IL-6, and IL-10 were tested by using ELISA kits in accordance with the manufacturer’s instructions (Nanjing Jiancheng Bioengineering Institute, Nanjing, China).

### 2.6. Real-Time Quantitative (RT-PCR)

For total RNA extraction, a sample of 50.0–60.0 mg of tissue (intestinal mucosa) was homogenized in 1.00 mL of RNAiso Plus (TaKaRa, Dalian, China). The RNA quality and quantity were then assessed using a Nano Drop ND-1000 (Nano Drop Technologies, Wilmington, DE, USA) of optical density at 260 and 280 nm. The concentration of RNA samples was diluted with diethyl pyrocarbonate-treated water to 0.5 μg/μL, then reverse-transcribed into cDNA using the Prime Script RT Master Mix reagentkit in accordance with the manufacturer’s instructions (TaKaRa, Dalian, China) immediately. The total RNA of the samples was processed to synthesize complementary DNA with a PrimeScript^TM^ RT reagent kit according to the manufacturer’s instructions (TaKaRa, Dalian, China). The reverse transcription was conducted at 37 ${}^{{}^{\circ}}$C for 15 min, 85 ${}^{{}^{\circ}}$C for 5 s. The relative mRNA expression of target genes was assessed using quantitative real-time PCR. The primer sequences, including IL-1β, TNF-α, IFN-γ, IL-4, IL-10, Occludin, Claudin-1, Claudin-2, ZO-1, β-actin, and their gene bank ID numbers are presented in Table 2.

### 2.7. Gut Microbiota Sequencing

Total genome DNA was isolated from the cecum using the QIAamp DNA Stool Mini Kit (QIAGEN, Dusseldorf, Germany), and DNA concentration and purity were measured on 1% agarose gels. The v3-v4 region of the 16S rDNA gene sequences were amplified by using 338F (ACTCCTACGGGAGGCAGCAG) and 806R (GGACTACHVGGGTWTCTAAT).Then, the elution with Tris-HCl buffer and detection by 2% agarose electrophoresis were done, and all amplicons were purified using an AxyPrep DNA gel extraction kit (Axygen Biosciences, Union City, CA, USA). After being quantified and purified, amplicons were sequenced on an Illumina MiSeq Sequencer. The data were all analyzed using the free online platform of Majorbio Cloud Platform (www.majorbio.com). The sequences were analyzed and assigned to operational taxonomic units (OTUs; 97% identity). Alpha diversity included calculation of ACE, Chao1, Shannon, and Simpson indices, beta diversity using principal coordinate analysis (PCoA) and unweighted Unifrac cluster tree and environmental factor correlation analysis.

## 3. Statistical Analysis

All data were normalized using SPSS 19.0 software and then subjected to statistical analysis using one-way analysis of variance (ANOVA). The replicate was defined as an experimental unit for the trail. The Tukey’s multiple comparison test was used to compare the differences among different groups. Data are expressed as the means ± standard error of the mean (SEM). The difference was considered to be significant at *P* < 0.05. An ANOVA test with *P* between 0.05 and 0.10 was considered as a trend toward significance.

## 4. Results

### 4.1. Intestinal Morphological Analysis

Table 3 showed that the diet with 500 mg/kg rutin significantly increased the villus height of jejunum (*P* < 0.05). Meanwhile, the diet with 500 mg/kg and 1000 mg/kg rutin significantly increase the ratio of villus height to crypt compared with the control group (*P* < 0.05). However, crypt depth of jejunum, as well as villus height, crypt depth, and the ratio of villus height to crypt depth of ileum were not altered in response to rutin treatments (*P* > 0.05).

### 4.2. Intestinal Barrier-Associated Gene Expression

As shown in Table 4, in the jejunal mucosa, a diet with rutin at 500 mg/kg enhanced claudin-2 expression (*P* < 0.05), but there was no difference in gene abundance of ZO-1, occludin, and claudin-1 among three groups (*P* > 0.05). In addition, a diet with 1000 mg/kg rutin had no effect on the expression of ZO-1, occludin, claudin-1, and claudin-2 in the jejunal mucosa (*P* > 0.05). Compared with the control group, dietary supplementation of rutin at 500 mg/kg and 1000 mg/kg had no significant effect on the expression of tight barrier genes (ZO-1, occludin, claudin-1 and claudin-2) in ileal mucosa (*P* > 0.05).

### 4.3. Intestinal Antioxidant Capacity

Table 5 showed that the two rutin supplemented diets had no significant influence on antioxidant parameters including MDA, SOD, and T-AOC (*P* > 0.05) compared with the control group in laying hens.

### 4.4. Intestinal Immunity

The results in Table 6 indicated that a diet with 500 mg/kg rutin supplementation raised the sIgA content in the jejunum mucosa (*P* < 0.05), Meanwhile, adding 1000 mg/kg rutin to the diet significantly increased the sIgA and IgM contents (*P* < 0.05). However, dietary supplementation of rutin had no significant effect on jejunal IgG content (*P* > 0.05). In the ileum mucosa, the contents of sIgA, IgG, and IgM significantly increased in response to a diet with 500 mg/kg rutin supplementation (*P* < 0.05), and a diet with 1000 mg/kg rutin supplementation significantly increased the contents of sIgA, IgG, and IgM compared with the control group, but had no difference on the contents of IgG compared with the 500 mg/kg supplementation group (*P* > 0.05).

In comparison to the control group, dietary supplementation of 500 mg/kg rutin significantly reduced the TNF-α content in the jejunal mucosa (*P* < 0.05), but had no significant effect on other cytokines (IL-1β, IFN-γ, IL-4 and IL-10) (*P* < 0.05). In comparison to the control group, dietary supplementation with 1000 mg/kg rutin lowered the level of IL-1β, TNF-α and IFN-γ in the jejunal mucosa (*P* < 0.05), while increasing the amount of IL-4 (*P* < 0.05), and there was no significant impact on IL-10 (*P* > 0.05). Dietary supplementation of 500 mg/kg rutin reduced the contents of TNF-α and IFN-γ in the ileal mucosa (*P* < 0.05) and raised the contents of IL-10 (*P* < 0.05), while there was no significant difference on IL-1β and IL-4 (*P* > 0.05). Diet treatment with 1000 mg/kg rutin considerably reduced the contents of IL-1β, TNF-α and IFN-γ in the ileal mucosa (*P* < 0.05), while significantly increasing the contents of IL-4 and IL-10 (*P* < 0.05). When compared to a diet with 500 mg/kg rutin supplementation, adding 1000 mg/kg rutin reduced the level of IL-1β and TNF-α significantly (*P* < 0.05), as shown in Table 7.

### 4.5. Intestinal Gene Expression of Cytokines

The results in Table 8 demonstrate that dietary supplementation of 500 mg/kg and 1000 mg/kg rutin significantly reduced the mRNA expression of IL-1β, IFN-γ, and IL-4 in the jejunal mucosa when compared to the control group (*P* < 0.05), whereas TNF-α and IL-10 mRNA expression levels were unaffected (*P* > 0.05). Both groups treated with rutin considerably lowered TNF-α mRNA expression while significantly increasing IFN-γ, IL-4 and IL-10 mRNA expression compared to the control group in ileal mucosa (*P* < 0.05) and the levels of IL-1β mRNA expression were unaltered (*P* > 0.05).

### 4.6. Cecal Microbiota

**Figure 1:**
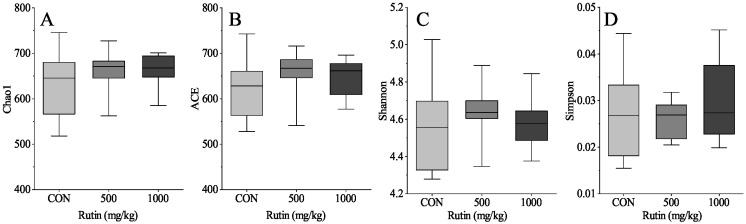
Alpha diversity of caecal microflora in aged laying hens. Chao1 (**A**), ACE (**B**), Shannon (**C**), and Simpson (**D**). Data are expressed as the mean ± SEM. Different lower-case letters indicate a significant difference among different groups (*P* < 0.05).

**Figure 2:**
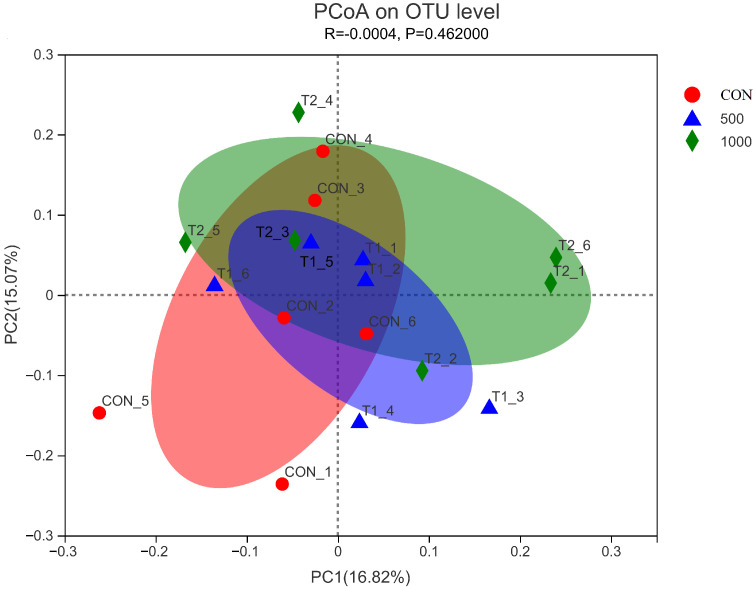
PCoA analysis of caecal microflora in aged laying hens.

**Figure 3:**
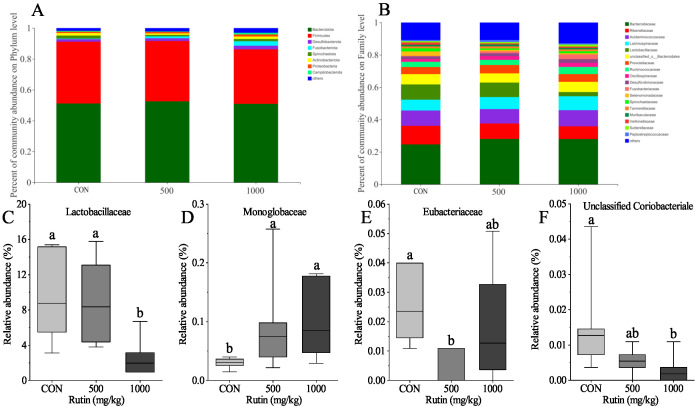
Beta diversity of caecal microflora composition in aged laying hens. (**A**,**B**) bar plots of microbial composition at phylum (**A**) and family (**B**) levels; (**C**–**F**), significantly differential bacteria taxa at family levels. Data are presented as mean ± SEM and analyzed by Kruskal-Wallis H test. Different lower-case letters indicate a significant difference among different groups (*P* < 0.05).

The results demonstrated that dietary rutin had no influence on the community richness (Chao1 and ACE) and the community diversity (Shannon and Simpson) indices in this research (*P* > 0.05) (Figure 1). Furthermore, PCoA demonstrated that a diet with rutin supplementation had no effect on the cecal microbiota composition of aged laying hens (*P* > 0.05) (Figure 2). Rutin supplementation had no effect on the relative abundances at the phylum level (Figure 3A). At the family level, when compared to control group, a diet with 500 mg/kg rutin supplementation raised the relative abundance of Monoglobaceae and decreased the relative abundance of Eubacteriaceae (*P* < 0.05) (Figure 3D,E). In the 1000 mg/kg rutin group, the relative abundance of Lactobacillaceae and Unclassified Coriobacteriale was considerably lower and the relative abundance of Monoglobaceae was higher than in the control group (*P* > 0.05) (Figure 3C,F).

## 5. Discussion

Intestinal integrity and intestinal morphology play a crucial role in intestinal function, such as the absorption of nutrient, and form a major physical barrier to protect against pathogens and toxic compounds [23,34]. An increase in the height or the width of the villus indicates an increase in the surface area, which can better absorb the available nutrients, thereby regulating the nutritional status and improving the development and health of the poultry [35]. Fideles et al. [36] pointed out that pretreatment with rutin (100 and 200 mg/kg) showed a marked reversal in villi shortening in the duodenum and the jejunum in mice of 5-Fluorouracil (FU) induced experimental intestinal mucositis; however, all rutin doses failed to reverse the villi shortening caused by 5-FU in the ileum. Additionally, Zhang et al. [37] reported that dietary rutin (250 mg/kg) and rutin (1000 mg/kg) in weaned mice for 21 days increased jejunum villus height, indicating that rutin improved intestinal morphology. The current study found that the villus height and villus-crypt ratio were longer in a rutin-supplemented diet in jejunal compared with the control group. Rutin had no influence on ileal villus height, crypt depth, and the villi-crypt ratio. Rutin has a greater impact on gut morphology in the jejunum than in the ileum. Previous research has suggested that rutin may reduce inflammation and oxidative stress via the cyclooxygenase 2 (COX-2) pathway, while also improving intestinal morphology [36]. However, the detailed mechanism of rutin on intestinal morphology needs further evaluation. The diet with 500 mg/kg rutin dramatically improved claudin-2 expression in the jejunum. Claudin-2 is expressed in the tight junctions of leaky epithelia, where it forms cation-selective and water permeable paracellular channels [38]. The results suggested that rutin is beneficial for improving intestinal integrity.

In vitro, phenolic compounds have ideal structural chemistry for free radical-scavenging activities, high levels of 2,2-diphenyl-1-picrylhydrazyl (DPPH) free radical scavenging activity, and have been shown in vitro to be more effective antioxidants than vitamins E and C on a molar basis [39,40]. In the animal antioxidant system, SOD removes superoxide radicals by accelerating their conversion to hydrogen peroxide [41]. MDA is a byproduct of polyunsaturated fatty acid peroxidation in cells, and MDA levels are routinely used to assess oxidative stress and antioxidant status [42]. T-AOC is usually applied as a comprehensive measure to assess an animal’s antioxidant capability [43]. A previous study have indicated that dietary rutin supplementation enhanced the serum antioxidant capacity by increasing SOD, CAT, and GSH-Px activity while decreasing MDA levels in serum of broilers [32]. In this study, rutin supplementation in the diet had no effect on the levels of MDA, and T-AOC, or SOD activity in the jejunum and ileum. This suggests that the antioxidant effect of rutin differs significantly in vivo and in vitro, and that differences in antioxidant ability of rutin in vivo studies may be related to animal species, age, tissue, and other factors.

The gut is the biggest immunological organ [44]. Secretory immunoglobulin A (sIgA) is a crucial line of defense on the mucosal surface of the intestine that protects the intestinal epithelium from toxins and pathogenic bacteria [45]. Our findings revealed that a diet with 500 mg/kg rutin increased the sIgA content but had no effect on the IgG and IgM content. Furthermore, a diet with 1000 mg/kg rutin can enhance sIgA and IgM concentrations. Ileal immunoglobulins were more responsive to rutin addition than those in the jejunum. The addition of two doses of rutin, 500 mg/kg and 1000 mg/kg, increased the concentrations of sIgA, IgM and IgG in the ileal mucosa. The results showed that rutin increases the function of the intestinal immune barrier by promoting the synthesis and release of immune globulin in the intestinal mucosa of aged laying hens. Previous investigations have reported that rutin inhibits the production of proinflammatory cytokines in microglia by lowering TNF-α and IL-1β levels in human neuroblastoma ( SH-SY5Y ) cells [46]. Furthermore, rutin supplementation 10 or 20 days after trimethyltin (TMT) injection reduced the TMT-induced elevation of mRNA expression levels of reactive microglia marker and pro-inflammatory cytokines in rats [47]. In this study, we discovered that supplementing with rutin reduces TNF-α in jejunal and ileal mucosa, the effect on IFN-γ in the ileum was greater than that in the jejunum, and high-dose rutin supplementation can significantly decrease the IL-1β, TNF-α, and IFN-γ and increase IL-10 content. The research showed that dietary rutin supplementation had an influence on intestinal cytokines, which was dose-dependent. The effect on the ileum is stronger than that on the jejunum, which may be associated with the intestinal metabolism mechanism of rutin. When compared to the control group, dietary supplementation of rutin substantially reduced IL-1β, IFN-γ, and IL-4 mRNA expression in the jejunal mucosa. In the ileal mucosa, both groups treated with rutin dramatically reduced TNF-α mRNA expression while significantly boosting IFN-γ, IL-4, and IL-10 mRNA expression. In view of the above data, we speculated that rutin can regulate cytokine content via modulating cytokine gene expression, and it can also function as an immunomodulator to reduce inflammation.

Gut bacteria play an important role in rutin function [48]. The majority of dietary rutin passes through the large intestine, where the colonic bacteria breaks down rutin into quercetin aglycone. Enterobacteria can then absorb or further degrade the quercetin aglycone to form different ring-fission products [49]. Rutin treatments all modulated the gut microbiota in rats during high diet fat intake [50]. The influence of rutin on the alpha and beta diversity of the gut microbiota in laying hens was not seen in this investigation. Rutin, on the other hand, increased the relative abundance of Lactobacillaceae. Lactobacillus has been demonstrated in several studies to increase the amount of Treg cells, which are key in controlling inflammation [51,52]. Meanwhile, rutin treatment reduced the relative abundance of Monoglobaceae, Eubacteriaceae, and Unclassified Coriobacteriale in the current research. Based on these findings, we infer that rutin may alter gut microbial composition and thereby change the intestinal immune system function in aged laying hens.

## 6. Conclusions

This study indicated that rutin supplementation for aged laying hens can modify the intestinal morphology and intestinal immune status, and modulate intestinal microbes in aged laying hens. The diet with 500 mg/kg of rutin showed an obvious effect on aged laying hens. Thus, a rutin supplementation of 500 mg/kg can act as a functional feed additive in aged laying hens to improve gut health and reduce inflammation.

## Figures and Tables

**Table 1:** Composition and nutrient level of basal diet (g/kg, as fed basis unless otherwise stated).

Item	Content
Ingredient	
Corn grain	640
Soybean meal	240
Limestone	90
Premix *	30
Calculated nutrient levels	
Apparent metabolizable energy (MJ/kg)	11.04
Crude protein	161.6
Lysine	8.0
Methionine + cystine	6.2
Calcium	36
Total phosphorus	5.6

* Supplied per kg of diet: Vitamin A, 10,000 IU; vitamin D3, 3000 IU; vitamin E, 20 IU; vitamin K3, 1.5 mg; vitamin B1, 2 mg; vitamin B2, 6 mg; vitamin B6, 3 mg; pantothenic acid, 10 mg; vitamin B12, 0.02 mg; nicotinamide, 40 mg; folic acid, 1 mg; biotin, 0.24 mg; choline chloride, 350 mg; Fe (ferrous sulfate), 80 mg; Cu (copper sulfate), 8 mg; Mn (manganese sulfate), 100 mg; Zn (zinc oxide), 65 mg; I (calcium iodate), 0.8 mg; Se (sodium selenite), 0.3 mg; calcium, 3.6 g; phosphorus, 2.4 g; methionine, 1 g; sodium chloride, 3 g.

**Table 2:** Sequences for real-time PCR primers.

Genes	Gene Bank ID		Primer Sequence (5${}^{\prime }$-3${}^{\prime }$)	Product Length (bp)
IL-1β	NM_204524.1	F	TGCCTGCAGAAGAAGCCTCG	204
		R	GACGGGCTCAAAAACCTCCT	
TNF-α	NM204267	F	TGTGTATGTGCAGCAACCCGTAGT	229
		R	GGCATTGCAATTTGGACAGAAGT	
IFN-γ	NM_205149.1	F	ATGTAGCTGACGGTGGACCT	196
		R	TTCACGCCATCAGGAAGGTT	
IL-4	NM_001007079.1	F	GTGCCCACGCTGTGCTTAC	82
		R	AGGAAACCTCTCCCTGGATGTC	
IL-10	NM_001004414.2	F	GGAGCTGAGGGTGAAGTTTGA	129
		R	GACACAGACTGGCAGCCAAA	
Occludin	NM_205128.1	F	CCGTAACCCCGAGTTGGAT	214
		R	ATTGAGGCGGTCGTTGATG	
Claudin-1	NM_001013611.2	F	AGGTGTACGACTCGCTGCTT	242
		R	AGCCACTCTGTTGCCATACC	
Claudin-2	NM_001277622.1	F	CCTGCTCACCCTCATTGGAG	144
		R	GCTGAACTCACTCTTGGGCT	
ZO-1	XM_015278975.2	F	TTTACATGATGACCGCCTGT	213
		R	GAATCCTCCCTAACGGGTTC	
β-actin	NM_205518	F	TGCTGTGTTCCCATCTATCG	150
		R	TTGGTGACAATACCGTGTTCA	

**Table 3:** Effect of rutin on the intestinal morphology of aged laying hens.

Items	Rutin, mg/kg			SEM	*P*-Value
	CON	500	1000		
Jejunum					
Villus height (μm)	935 ${}^{b}$	1100 ${}^{a}$	981 ${}^{a,b}$	29	0.044
Crypt depth (μm)	142	132	119	5	0.175
Villus height: crypt depth	6.7 ${}^{b}$	8.6 ${}^{a}$	8.5 ${}^{a}$	0.3	0.003
Ileum					
Villus height (μm)	675	791	808	36	0.271
Crypt depth (μm)	150	151	170	5	0.278
Villus height: crypt depth	4.7	5.5	5.0	0.2	0.259

^a,b^ Means with different superscripts within a row differ significantly (*P* < 0.05). One-way ANOVA was used to analyze the data (means ± SEM).

**Table 4:** Effects of rutin supplementation on the relative mRNA abundances of tight junction in the intestine of aged laying hens.

Items	Rutin, mg/kg			SEM	*P*-Value
	CON	500	1000		
Jejunum					
ZO-1	1.00	0.91	1.04	0.09	0.832
Occludin	1.00	0.80	0.81	0.06	0.321
Claudin-1	1.00	1.49	1.38	0.18	0.513
Claudin-2	1.00 ${}^{b}$	2.06 ${}^{a}$	1.04 ${}^{b}$	0.16	0.002
Ileum					
ZO-1	1.00	1.27	1.28	0.09	0.346
Occludin	1.00	1.70	1.52	0.12	0.050
Claudin-1	1.00	0.74	1.16	0.11	0.313
Claudin-2	1.00	1.46	1.63	0.20	0.458

^a,b^ Means with different superscripts within a row differ significantly (*P* < 0.05). One-way ANOVA was used to analyze the data (means ± SEM).

**Table 5:** Effect of rutin on the intestinal antioxidant status of aged laying hens.

Items	Rutin, mg/kg			SEM	*P*-Value
	CON	500	1000		
Jejunum					
MDA (nmol/mg protein)	0.49	0.45	0.39	0.02	0.194
SOD (U/mg protein)	180.03	181.26	175.11	4.59	0.861
T-AOC (U/mg protein)	1.37	1.37	1.30	0.06	0.889
Ileum					
MDA (nmol/mg protein)	0.38	0.41	0.38	0.08	0.736
SOD (U/mg protein)	176.10	173.84	186.30	4.70	0.543
T-AOC (U/mg protein)	1.20	1.29	1.43	0.05	0.116

MDA: malondialdehyde; SOD: superoxide dismutase; T-AOC: total antioxidant capacity. One-way ANOVA was used to analyze the data (means ± SEM).

**Table 6:** Effect of rutin supplementation on the intestinal immunoglobulins levels of aged laying hens.

Items	Rutin, mg/kg			SEM	*P*-Value
	CON	500	1000		
Jejunum					
sIgA (μg/mg)	1.11 ${}^{b}$	1.41 ${}^{a}$	1.60 ${}^{a}$	0.06	0.000
IgG (μg/mg)	167.06	196.45	198.78	6.28	0.062
IgM (μg/mg)	1.98 ${}^{b}$	2.38 ${}^{a,b}$	2.82 ${}^{a}$	0.11	0.003
Ileum					
sIgA (μg/mg)	1.10 ${}^{c}$	1.48 ${}^{b}$	1.97 ${}^{a}$	0.09	0.000
IgG (μg/mg)	169.59 ${}^{b}$	208.81 ${}^{a}$	229.10 ${}^{a}$	8.67	0.008
IgM (μg/mg)	1.79 ${}^{c}$	2.37 ${}^{b}$	3.02 ${}^{a}$	0.15	0.000

^a,b,c^ Means with different superscripts within a row differ significantly (*P* < 0.05). One-way ANOVA was used to analyze the data (means ± SEM).

**Table 7:** Effect of rutin supplementation on the levels of intestinal cytokines of aged laying hens.

Items	Rutin, mg/kg			SEM	*P*-Value
	CON	500	1000		
Jejunum					
IL-1β (ng/g)	61.60 ${}^{a}$	55.81 ${}^{a}$	46.41${}^{b}$	1.95	0.001
TNF-α (ng/g)	24.93 ${}^{a}$	20.00 ${}^{b}$	15.13 ${}^{c}$	1.15	0.000
IFN-γ (pg/g)	298.34 ${}^{a}$	256.01 ${}^{a,b}$	225.25 ${}^{b}$	10.20	0.005
IL-4 (pg/g)	188.97 ${}^{b}$	224.3 ${}^{a,b}$	258.12 ${}^{a}$	10.41	0.014
IL-10 (ng/g)	12.29	17.89	17.57	1.27	0.127
Ileum					
IL-1β (ng/g)	58.04 ${}^{a}$	53.29 ${}^{a}$	47.53 ${}^{b}$	1.34	0.001
TNF-α (ng/g)	24.37 ${}^{a}$	18.01 ${}^{b}$	13.50 ${}^{c}$	1.25	0.000
IFN-γ (pg/g)	294.07 ${}^{a}$	241.02 ${}^{b}$	208.40 ${}^{b}$	10.88	0.001
IL-4 (pg/g)	185.27 ${}^{b}$	231.52 ${}^{a,b}$	233.51 ${}^{a}$	8.90	0.032
IL-10 (ng/g)	11.72 ${}^{b}$	18.41 ${}^{a}$	23.10 ${}^{a}$	1.35	0.000

^a,b,c^ Means with different superscripts within a row differ significantly (*P* < 0.05). One-way ANOVA was used for analysing the data (means ± SEM).

**Table 8:** Effects of rutin supplementation on the relative mRNA abundances of the intestinal cytokines of aged laying hens.

Items	Rutin, mg/kg			SEM	*P*-Value
	CON	500	1000		
Jejunum					
IL-1β	1.00 ${}^{a}$	0.32 ${}^{b}$	0.43 ${}^{b}$	0.10	0.004
TNF-α	1.00	0.85	0.87	0.08	0.741
IFN-γ	1.00 ${}^{a}$	0.29 ${}^{b}$	0.41 ${}^{b}$	0.09	0.000
IL-4	1.00 ${}^{a}$	0.25 ${}^{b}$	0.33 ${}^{b}$	0.12	0.015
IL-10	1.00	1.02	1.06	0.03	0.785
Ileum					
IL-1β	1.00	0.93	1.12	0.05	0.312
TNF-α	1.00 ${}^{a}$	0.66 ${}^{b}$	0.70 ${}^{b}$	0.05	0.005
IFN-γ	1.00 ${}^{b}$	1.46 ${}^{a}$	1.42 ${}^{a}$	0.07	0.006
IL-4	1.00 ${}^{b}$	1.88 ${}^{a}$	1.55 ${}^{a}$	0.11	0.000
IL-10	1.00 ${}^{b}$	1.32 ${}^{a}$	1.27 ${}^{a}$	0.05	0.004

^a,b^ Means with different superscripts within a row differ significantly (*P* < 0.05). One-way ANOVA was used to analyze the data (means ± SEM).

## Data Availability

Data is contained within the article.
